# Deep data mining reveals variable abundance and distribution of microbial reproductive manipulators within and among diverse host species

**DOI:** 10.1371/journal.pone.0288261

**Published:** 2023-07-11

**Authors:** Paloma Medina, Shelbi L. Russell, Russell Corbett-Detig

**Affiliations:** Genomics Institute, Department of Biomolecular Engineering UC Santa Cruz, Santa Cruz, CA, United States of America; University of Iceland, ICELAND

## Abstract

Bacterial symbionts that manipulate the reproduction of their hosts are important factors in invertebrate ecology and evolution, and are being leveraged for host biological control. Infection prevalence restricts which biological control strategies are possible and is thought to be strongly influenced by the density of symbiont infection within hosts, termed titer. Current methods to estimate infection prevalence and symbiont titers are low-throughput, biased towards sampling infected species, and rarely measure titer. Here we develop a data mining approach to estimate symbiont infection frequencies within host species and titers within host tissues. We applied this approach to screen ~32,000 publicly available sequence samples from the most common symbiont host taxa, discovering 2,083 arthropod and 119 nematode infected samples. From these data, we estimated that *Wolbachia* infects approximately 44% of all arthropod and 34% of all nematode species, while other reproductive manipulators only infect 1–8% of arthropod and nematode species. Although relative titers within hosts were highly variable within and between arthropod species, a combination of arthropod host species and *Wolbachia* strain explained approximately 36% of variation in *Wolbachia* titer across the dataset. To explore potential mechanisms for host control of symbiont titer, we leveraged population genomic data from the model system *Drosophila melanogaster*. In this host, we found a number of SNPs associated with titer in candidate genes potentially relevant to host interactions with *Wolbachia*. Our study demonstrates that data mining is a powerful tool to detect bacterial infections and quantify infection intensities, thus opening an array of previously inaccessible data for further analysis in host-symbiont evolution.

## Introduction

Bacterial symbionts of eukaryotic hosts are ubiquitous in nature, but can be challenging to detect and quantify. Included among these symbionts are intracellular bacteria, called reproductive manipulators (RM), that alter host reproduction in order to increase their likelihood of vertical transmission to the next host generation [1–4]. Many of these reproductive manipulations are positively correlated with within-host symbiont density and have the potential to drive symbionts, along with infected host genotypes, to high frequencies in host populations [5–10]. In addition to these frequency-increasing mechanisms, many RM strains are capable of horizontally transferring from an infected host to an uninfected host, resulting in the spread of these symbionts among host species [11]. Post-infection, the cellular cost of growing symbionts and the reliability of vertical transmission also impact host infection frequencies [12–14]. Because these bacteria are so intimately woven into their hosts’ biologies, infections must be confirmed with molecular approaches, making population and community-wide assessments laborious and costly.

The impressive reproductive manipulation abilities of these bacteria have recently been leveraged to control problematic host populations, such as Dengue virus-harboring mosquitoes [15–19]. RM strains have different impacts on host biology and host reproduction, which can be used to 1) reduce host population sizes (*e*.*g*., through the sterile male technique [20, 21], 2) drive symbiont infection frequencies to high levels for some other phenotypic benefit (*e*.*g*., viral inhibition [15, 17–19], or 3) kill or render the host infertile (*e*.*g*., in obligate infections [22]). Importantly, different reproductive manipulator strains can have incompatible rescue abilities [23–25]. Given that these strategies rely on knowing the infection status of host populations and the identity of infecting strains, it is necessary to characterize the natural diversity and distribution of these bacteria among arthropod and nematode hosts.

Quantifying the density of symbiont cells within host tissues, termed titer, has been challenging historically, despite its importance in understanding the strength of reproductive manipulation, reliability of vertical transmission, and rate of novel infections. Reliable within-host symbiont quantification requires a sensitive and calibratable read-out, such as fluorescence intensity in imaging data, amplicon counts in qPCR, or read counts in sequencing-based approaches (e.g., [26]). Thus, the accessible, but binary results of PCR-based detection assays are suitable for frequency estimation, but not titer. Much of what we know about titer has been extrapolated from a few well-studied systems [7, 27, 28]. Indeed, a recent study investigated titer using a bioinformatic approach, but was limited to a single symbiont species [29]. A symbiont must be present at sufficiently high frequencies within a host to promote successful transmission to subsequent host generations [8–10, 30] without being so abundant as to impose a significant fitness cost on the host [7, 27, 31, 32]. This titer homoeostasis must be balanced against variation induced by the environment and host diet [31]. Given how difficult titer is to measure and how variable it can be, little is known about the relative abundances of reproductive manipulators within and among individuals of a host species, or across bacterial strains and species.

*Wolbachia pipientis* (Alphaproteobacteria) is one of the best studied reproductive manipulators due to its wide distribution [33–35], presence in model organisms such as *D*. *melanogaster* [36], and use in biological control. Estimates of the frequency of *Wolbachia* infections amongst arthropod species range from 11% [37] to 76% [38]. The large variation in estimates is likely an effect of methodological differences including sampling bias and variation in PCR assay sensitivity. Other reproductive manipulators in the *Cardinium* (Sphingobacteriia), *Arsenophonus* (Gammaproteobacteria), *Rickettsia* (Alphaproteobacteria), and *Spiroplasma* (Mollicutes) genera are reported to occur in 4% to 7% of all species [39]. However, these estimates are even less certain than those for *Wolbachia* because fewer data are available for these clades, and little is known about the within-host titer of these symbionts. Undersampling imposes a barrier to confidently detect low frequency infections because the probability of sampling an infected individual is positively correlated with the prevalence of the symbiont and the number of individuals sampled from the host population.

Whole genome sequencing and bioinformatic approaches offer appealing alternatives to conventional PCR-based survey methods to simultaneously estimate reproductive manipulator infection rates and within-host titer [29, 37]. These methods are not biased by primer selection, may be less sensitive to false positives due to contamination, and enable testing of large numbers of samples [40]. When the genome of an individual is sequenced with Illumina shotgun sequencing, any potential symbionts are also sequenced. This makes the publicly-available databases a treasure trove for sampling reproductive manipulators with bioinformatic approaches [29, 37, 41–43]. In fact, as of 2020 the NCBI Sequence Read Archive (SRA) [44] contained more than 60,000 sequencing runs for samples tagged as Arthropoda alone. Searching, or mining, genomic sequencing data has been shown to be a cost effective and powerful strategy to detect *Wolbachia* infections [37, 41]. However, prior studies only include few reproductive manipulator species [29, 37, 41, 43] or were focused on a single host species [41]. Indeed, one of the most compelling arguments for using non-targeted, publicly available data is to mitigate ascertainment biases away from selecting species already known to harbor reproductive manipulator infections.

Here, we establish and validate a computational pipeline to determine the infection status of host samples downloaded from the NCBI Sequence Read Archive (SRA) database and estimate the titer of symbionts within positively infected host individuals. We use this approach to (i) quantify *Wolbachia* infection frequency and infection titer of hosts, (ii) quantify the infection frequency and titer of other reproductive manipulator symbiont clades, (iii) compare infection frequencies and titers (from i and ii) among host groups, and (iv) identify *D*. *melanogaster* SNPs associated with *Wolbachia* titer. From this work, we classified 2,083 arthropod and 119 nematode samples as infected with a reproductive manipulator, including 93 species with previously unknown infections. We show substantial variation in symbiont titer within and between arthropod host species which is only partially explained by the combination of species and strain. We present extensive validation of our methods, including orthogonal *in vivo* approaches, which confirm that mining public databases is a highly accurate and sensitive approach for detecting reproductive manipulator bacterial infections at the genomic level.

## Results and discussion

### Validation of bacterial detection pipeline

**Fig 1:**
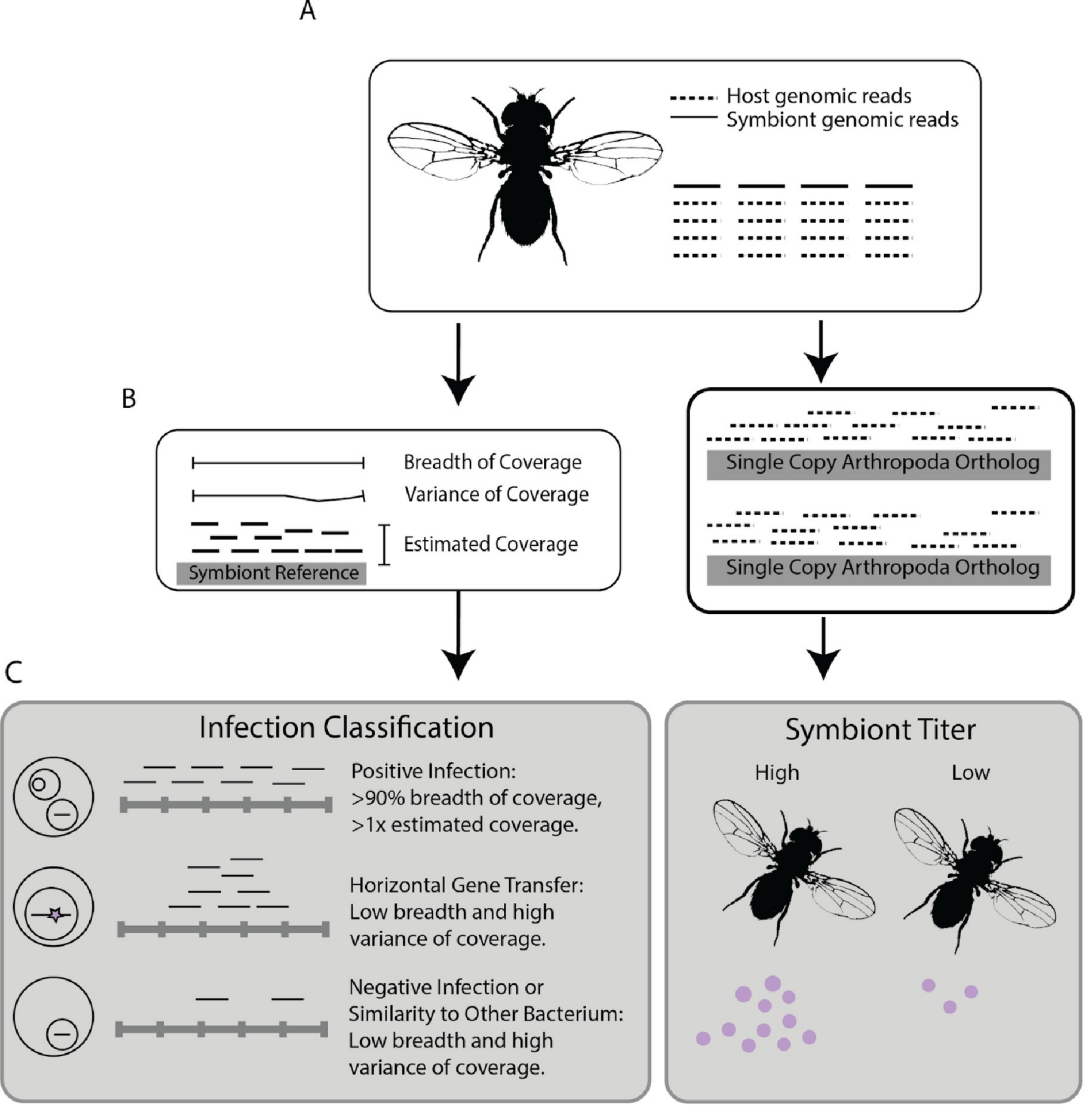
A schematic of the computational pipeline used to determine the reproductive manipulator infection status and symbiont titer of a sequencing run. The pipeline **(A)** takes in a sample’s unique identification number, then downloads two million reads (includes symbiont and host genomic reads). Then, **(B)** reads are aligned to *Wolbachia*, *Arsenophonus*, *Spiroplasma*, *Cardinium*, and *Rickettsia* reference genomes. Also, reads are aligned to a set of 1066 single copy ancestral orthologs obtained from ORTHODB v9 to estimate host coverage without requiring a reference genome. **(C)** Summary statistics for sample reads aligned to each reference are computed. If a sample had between 0.1 and 0.9 breadth of coverage, the full dataset was downloaded and the workflow repeated to prevent false negative calls. We apply coverage breadth and depth cutoffs to classify infection status as positive, or negative. To estimate symbiont titer, we compare the depth of coverage of host reads mapped to a set of single copy orthologs, to the coverage of symbiont reads mapped to a symbiont reference genome. Please see the methods section for more details on the approach for classification and titer computation.

We developed a powerful bioinformatic pipeline to identify reproductive manipulator infections and infection densities within Illumina sequencing datasets. Briefly, the approach compares sequencing reads from individual samples to a set of 102 reference genomes, representing the sampled diversity of reproductive manipulator bacterial species, to determine if a given host is positively infected (Fig 1, see Methods). We extensively characterized the sensitivity and accuracy of this bioinformatic pipeline using previously known *Wolbachia* infection statuses of the *Drosophila* Genetic Reference Panel (DGRP [45, 46]), samples known to harbor genetically divergent *Wolbachia* [47], and compared our method to previous studies.

### *Wolbachia* infection status of the DGRP and subsampling efficacy

To estimate the sensitivity and the specificity of our symbiont classification method, we compared our results to a previous extensive survey conducted by [41], which was PCR validated. We determined the reproductive manipulator infection status of 158 individuals from the Drosophila Genetics Research Panel (DGRP) [46] using our computational pipeline. Subsampling two million reads from each DGRP sequencing run generated no false positive nor false negative infection statuses (S1 Table). When sampling all reads instead, we found similar low error rates (S2 Table). This indicates our approach may be conservative for calling infections when read numbers are limited.

#### Effect of divergent reference genomes

Given that there may be unsampled genomic diversity among reproductive manipulator strains in nature, and this may bias the detection method, we characterized the method’s sensitivity to detecting divergent *Wolbachia* strains by comparing mapping results for a sample among diverse reference genomes (S1 and S2 Figs in S1 File). In addition to wMel, we used reference *Wolbachia* strains from host *Culex quinquefasciatus*, *Onchocerca ochengi*, *Brugia malayi*, *Cimex lectularius*, and *Pratylenchus penetrans* from *Wolbachia* Supergroup B, C, D, F, and L, respectively. When compared to wMel, these genomes exhibit 5–15% pairwise sequence divergence within alignable regions. Impressively, the method never produces false positives when determining infection status across all references using two million reads per sequencing run (S1 and S2 Tables).

False negative rates are similarly modest for all divergent *Wolbachia* references, at a maximum of 0–6% when aligning all reads in all but the most divergent reference genome (S1 Table). Across all arthropod samples, we expect most of the symbionts within these samples to have the most similarity to *Wolbachia* from supergroups A and B (wMel and wPip) [48–50]. So, finding that these strains can still be detected when using a nematode *Wolbachia* strain reference suggests that the method is very sensitive and the majority of *Wolbachia* infections were detected. Accuracy data for each reference genome are available in S1 and S2 Tables. Taken together, these data indicate that subsampling sequencing read data is a computationally efficient method to determine reproductive manipulator infection statuses of the majority of host samples (*i*.*e*., 97.5% can be accurately classified using a subsample of two million reads).

#### Comparison to other methods

A previous study by [37] used a related bioinformatic pipeline to screen arthropod individuals for *Wolbachia* infection. The key difference between our method and theirs is that they selected only three loci (*wsp*, *ftsZ*, and *groE* operon sequences) rather than complete genomes, and required extremely similar blast hits to categorize samples as positive (98 bp BLAST length matches at > = 98% identity to one or more of the reference genes and three or more matching sequence reads). They screened 2,545 arthropod sequencing runs, a subset of what we consider here, and found 173 (6.8%) of them were candidates for a positive *Wolbachia* infection which composed 11.8% of species tested. Their estimate ranges on the lower spectrum of *Wolbachia* frequency estimates. Therefore, we ran our classifier on sequencing runs that Pascar’s method determined to be uninfected in order to compare the sensitivity of their method using our whole genome approach.

Of the 2365 runs classified as negative for *Wolbachia* infection, we found our method classified 22 sequencing runs as positive (S3 Table). Considering the median breadth of coverage and estimated coverage depth was 0.98 and 11x, respectively, we are confident that these sequencing runs are strong candidates for positive *Wolbachia* infection. If we are to consider these as positive samples, we estimate that the false negative rate of Pascar and Chandler’s method is 11%. Given that their approach is relatively conservative in identifying positive infections, it is unsurprising that we find a low proportion of false positives, which at 1.7%, is similar to our own.

#### Identifying divergent *Wolbachia* strains

To further confirm the accuracy of our approach across a broader phylogenetic sampling of *Wolbachia* strains, we estimated the infection status for known positive infections of *Zootermopsis nevadensis* (termite), *Ctenocephalides felis* (flea), *Folsomia candida* (springtail), and *Osmia caerulescens* (solitary bee) using the BLAST-based approach. All of the sequencing runs were previously determined to harbor *Wolbachia* infections [47] and classified into supergroup H, B, E, and F, respectively. Importantly, some of these divergent supergroups are not represented in the reference database and therefore represent the most challenging cases for accurate detection using our pipeline. All host samples were found to be positive for *Wolbachia* infection except for a sample from supergroup H, even after running the pipeline on all the reads from that sample (S4 Table). These results suggest that this method can detect *Wolbachia* from genetically divergent supergroups, but may not detect all of *Wolbachia’s* genomic diversity. Future efforts may mitigate this challenge by incorporating an increasingly diverse array of reproductive manipulator reference genomes.

We note that other reproductive manipulators may also pose other challenges for accurate quantification beyond the specifics that we encountered in *Wolbachia* (*e*.*g*., *Cardinium*). However, in the absence of a high quality validation set and numerous true positives which are available for *Wolbachia*, it is challenging to formally evaluate this pipeline’s performance in the other reproductive manipulators. Nonetheless, the overall robustness and accuracy when applied to *Wolbachia* positive and negative controls, and the consistency of frequency and titer estimates with previous results suggests that there are few significant biases associated with our approach.

### Reproductive manipulators infect arthropod and nematode hosts in the SRA

Using our bioinformatic classification pipeline, we tested nearly all arthropod and nematode samples in the SRA database that had been whole genome shotgun sequenced (as of January 22, 2020). We found 240 arthropod species and 8 nematode species had samples infected with a reproductive manipulator out of 1,299 and 128 species tested, respectively (Table 1, S3 and S4 Figs in S1 File, S5 and S6 Tables, S1–S3 Data). Among these species, we identified 93 arthropod species with previously unreported reproductive manipulator infections (S7 Table).

**Table 1:** Raw infection counts of *Wolbachia*, *Spiroplasma*, *Rickettsia*, *Cardinium* and *Arsenophonus* infecting arthropods and nematodes. The frequency of positive samples or species is listed in parentheses.

	Samples		Species	
	Arthropoda	Nematoda	Arthropoda	Nematoda
**Total**	27256	5229	1299	128
**Infected With Any RM**	2083 (0.08)	119 (0.02)	240 (0.18)	8 (0.06)
**Infected With *Wolbachia***	1978 (0.07)	119 (0.02)	179 (0.14)	8 (0.06)

*Wolbachia* was the most frequent reproductive manipulator in arthropod samples and species (1,978 samples, 179 species, Table 1). We also found numerous arthropod species infected with other clades of reproductive manipulators (S5 Table). Specifically, *Cardinium*, *Rickettsia*, *Spiroplasma*, and *Arsenophonus* infect two, 54, 69, and nine arthropod species in the dataset, respectively. The substantially lower infection rates for other reproductive manipulators than for *Wolbachia* are consistent with prior work [51–53] (S5 Table, S3 Fig in S1 File).

*Wolbachia* was the sole reproductive manipulator found in nematodes besides an instance of *Cardinium*, as expected based on previous work [39, 54]. Almost all nematode species infected with *Wolbachia* are filarial worms (S6 Table), which is a result supported by earlier studies showing that filarial nematodes and *Wolbachia* are in an obligatory, mutualistic relationship [55–57]. There was one non-filarial nematode species infected with *Wolbachia*: *Pratylenchus penetrans*, a plant-parasitic nematode (order Tylenchida). This species has been shown previously to be infected with *Wolbachia* [54, 58]. These results, in addition to our validation experiments, indicate that our approach can accurately detect symbiont infections. Indeed, our survey does not distinguish between hosts infected with *Wolbachia* and endoparasites infected with *Wolbachia* that may infect hosts. Downstream functional annotation and analysis of endoparasitoids and symbionts would be necessary to determine the source of symbiont infection. This possibility may possibly explain specific infections, but likely does not explain the majority of the data.

We analyzed co-infections at two different levels: host species harboring samples infected with more than one reproductive manipulator clade and single samples infected by more than one bacterial clade. We found high rates of co-infections of different reproductive manipulators among arthropod host species. Species co-infections occurred in 29 of the 240 arthropod species that harbored any infection (*p* < 0.001 permutation test, see Methods, S5 Fig in S1 File, and S8 Table). The observed rate of co-infection is consistent with previous work that identified eight out of 44 species were co-infected ([39], *p* = 0.34, Fisher’s Exact Test). These results may indicate that a subset of host species are more likely to acquire novel symbionts than others or that they are simultaneously permissive to multiple symbiont types. This is supported by the observation that some host species harbor genetic variation that influences symbiont titer within host individuals [7, 59]. Alternatively, any direct or indirect mutualism between symbionts might facilitate the build-up of reproductive manipulators within a single species or host maternal lineage. In contrast, we found no evidence for an excess of individual samples infected by more than one reproductive manipulator relative to expectations obtained by permutation (*p* = 0.155, 27 individuals, S6 Fig in S1 File). This may suggest that competition between mixed strain infections might resolve faster than can be sampled. Indeed it is difficult to predict the impacts of mixed strains within single individuals, and outcomes (e.g. host lifespan) can vary substantially between symbiont strains and host species [60–63].

### Proportion of symbiont-infected arthropod species estimated to be 1–44%

We used a beta-binomial distribution to estimate the total proportion of species infected with a reproductive manipulator from the raw counts of infected SRA samples and species (see Methods). This approach accounts for variable sampling intensities (S5 Table) across host taxa and naturally low infection frequencies in some species [34]. Using this approach, we estimated that 44% (95% CI 29–61%), and 34% (95% CI 5–69%), of arthropod and nematode species, respectively, are infected with *Wolbachia* (Table 2). We note that these values are consistent with expectations from previous work [33–35, 38, 49, 64–69]. Moreover, because the SRA has been populated with samples mostly without considering *Wolbachia* infection status, our approach should provide a relatively unbiased estimate of global infection frequency. Nonetheless, it is possible that other sampling biases, *e*.*g*., medical relevance of focal species, might impact our estimates if medically relevant groups have unusual infection frequencies relative to an “average” arthropod or nematode. Additionally, metadata errors such as misspecified host species could contribute to both false positive and negative results.

**Table 2:** Estimated infection frequencies and confidence intervals from the data for *Wolbachia*, *Spiroplasma*, *Rickettsia* and *Arsenophonus* infecting arthropods and nematodes. All species in the dataset were downsampled to a maximum 100 individuals for frequency inference. We used a minimum infection frequency of 0.001 to classify a species as positively infected (See Methods).

Host Phylum	Reproductive Manipulator	2.5% CI	97.5% CI	Median	Mean
Arthropods	*Arsenophonus*	0.00	0.05	0.01	0.01
	*Rickettsia*	0.01	0.08	0.04	0.04
	*Spiroplasma*	0.03	0.16	0.08	0.08
	*Wolbachia*	0.29	0.62	0.44	0.44
Nematodes	*Wolbachia*	0.05	0.69	0.33	0.34

Among the other reproductive manipulators, we estimated *Arsenophonus*, *Rickettsia*, and *Spiroplasma* infected 1% (95% CI 0–5%), 3% (95% CI 1–8%), and 8% (95% CI 3–15%) of arthropod species, respectively (Table 2). We were not able to estimate global infection rates of *Cardinium* because of the extremely low rate of positive infection in the dataset. Owing to the fact that we do not have positive and negative controls readily available for each of these other reproductive manipulator clades, it is difficult to completely rule out infections that failed to map to the known reference(s) for each group and therefore induce a higher rate of false negatives. However, the results from mapping *Wolbachia* reads to extremely diverse reference genomes (e.g., 5–15% divergence) suggests that the rate of false negatives is low, provided the divergence within these other bacterial groups does not exceed the tested values and, as we note above, our raw frequency estimates are in line with previous work based on other methods (S4 Table).

### Taxonomic distribution of reproductive manipulator infections

**Fig 2:**
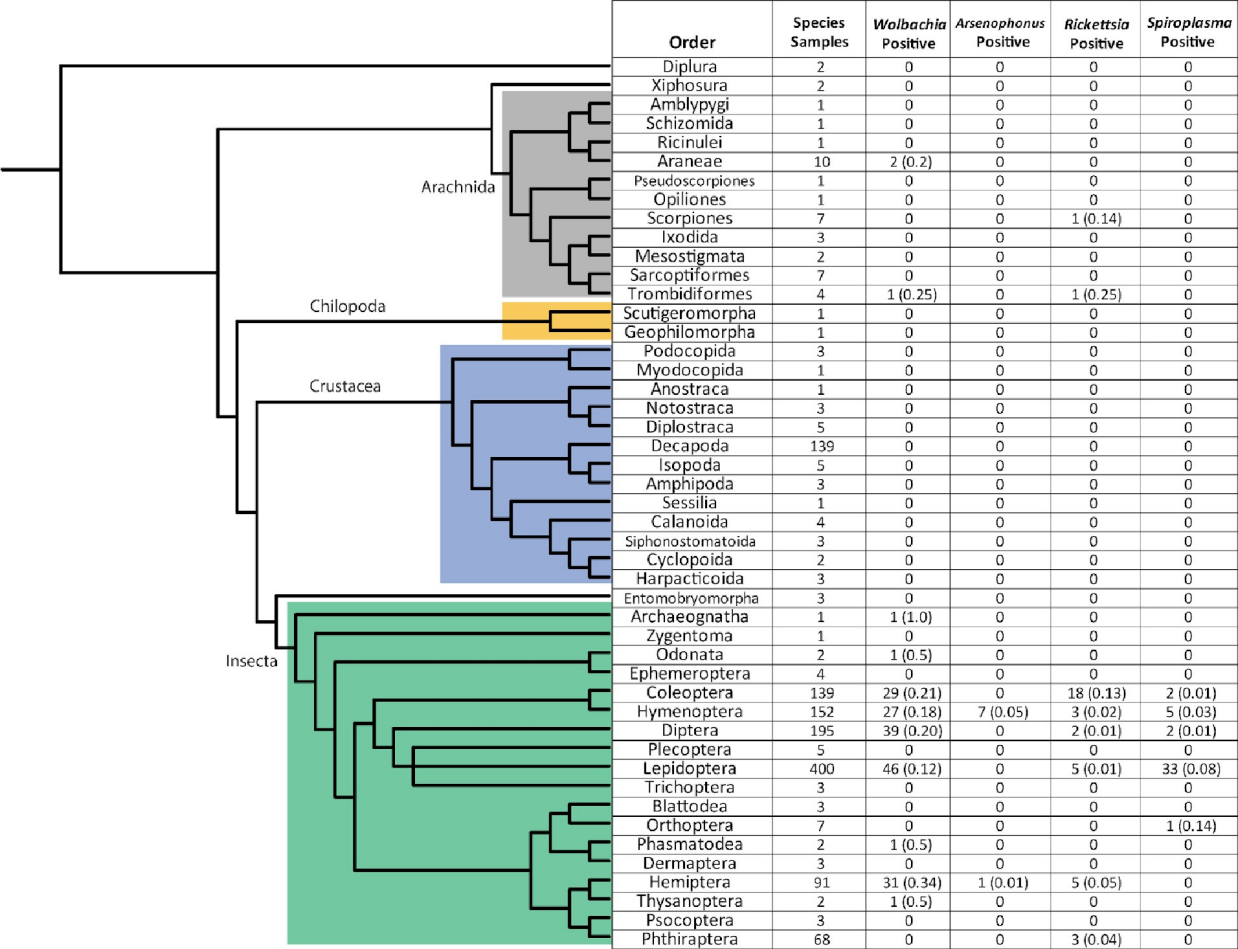
Phylogeny of Arthropoda orders tested and number of reproductive manipulator positive species within each order. The frequency of reproductive manipulator-positive species listed in parentheses. No frequency is listed if there was no infection within an arthropod order. We used the Tree of Life taxonomic and phylogenetic package and rotl [74], to group host species by their orders. We labeled arthropod clades containing two or more taxa with subphylum or class.

The taxonomic distribution of *Wolbachia* infections spans 11 arthropod orders, out of the 47 tested (Fig 2). Across all of the arthropod species that we studied here, the orders with the greatest number of species sampled are Hymenoptera, Diptera, Lepidoptera, Coleoptera, and Hemiptera. These orders had 152, 195, 400, 139, and 91 species sampled, respectively. Positively infected samples are dispersed widely across the phylogeny, including hosts as distant as Coleoptera and Araneae. Horizontal transmission among species contributes towards explaining *Wolbachia’s* large taxonomic distribution, despite only modest sequence level divergence among *Wolbachia* strains [50, 70–73].

*Wolbachia* infection frequencies vary substantially across insect orders. For example, Hemiptera, Hymenoptera, Diptera, and Coleoptera vary by approximately 30%, with 54%, 42%, 63%, 78% of species estimated to be infected, respectively (Table 3). We estimated the lowest *Wolbachia* infection frequency for Lepidoptera (36%, 95% CI 9.28–70.4%, Table 3). Lepidoptera’s infection frequency, in particular, may be slightly lower than the frequencies found in other orders of insects (*p* < 0.08, permutation test).

**Table 3:** *Wolbachia* global infection frequencies and confidence intervals generated for arthropod orders. All species in the dataset were downsampled to a maximum 100 individuals. Confidence intervals were generated using 1000 bootstrap replicates fitting a beta-binomial model to species infection frequency data among orders. A minimum infection frequency of 0.001 was used to classify a species as positively infected (See Methods).

Order	Mean	Median	2.50% CI	97.50% CI
Coleoptera	0.78	0.79	0.53	0.96
Diptera	0.63	0.64	0.30	0.90
Hymenoptera	0.42	0.41	0.12	0.77
Hemiptera	0.55	0.51	0.17	1.00
Lepidoptera	0.36	0.35	0.09	0.70

### Comparative study of titer across symbiont taxa

Bacterial symbiont density within a host, or titer, may be important for both host and symbiont fitness, but little is known about the relative abundances of reproductive manipulators within individuals of a host species, or across bacterial strains and species. Variation in titer may be a consequence of genetics (including host, symbiont, and combined host-symbiont genotype interactions) or a result of other biological variation among genetically similar individuals (*e*.*g*., due to temperature and diet [31, 75–77]).

Here, we use whole genome shotgun SRA datasets to estimate titer from the ratio of symbiont genome complements to host genome complements (see Methods). We used a reference panel consisting of 137 symbiont references to estimate symbiont genome complements and a panel of single copy orthologs to estimate host genome complements (S11 Table). We evaluated the accuracy of our approach using *in silico* and *in vivo* approaches. First, we found titer estimates using our bioinformatic approach were reflected in *in vivo* estimates of wRi and wMel, which are two of the most commonly studied *Wolbachia* strains (S7 Fig in S1 File). Second, we analyzed our approach’s accuracy to computing host genome coverage in arthropod samples. We found titer estimates of our approach to be consistent with a reference based approach (Spearman’s rho = 0.81, S8 Fig in S1 File). Third, we analyzed how titer estimates changed when choosing a more distant symbiont reference to compute titer. We would expect symbiont coverage estimates to vary significantly between references if titer estimates were impacted by the distance to the reference selected. We found our method was robust in estimating stable symbiont genome coverages (S9 Fig in S1 File, S9 Table). Lastly, we compared our approach to a reference-based approach to estimate titer in DGRP samples and found the two methods to yield similar results (Spearman’s rho = 0.95, S10 Fig in S1 File). These results suggest that our *in silico* approach can yield information about *Wolbachia* abundance in tissues that are most relevant to understanding *Wolbachia* transmission (i.e., the female germline) and that our method is robust in estimating stable symbiont genome coverages especially from *Wolbachia* Supergroups A and B, of which comprise the majority of tested arthropod infections.

**Fig 3:**
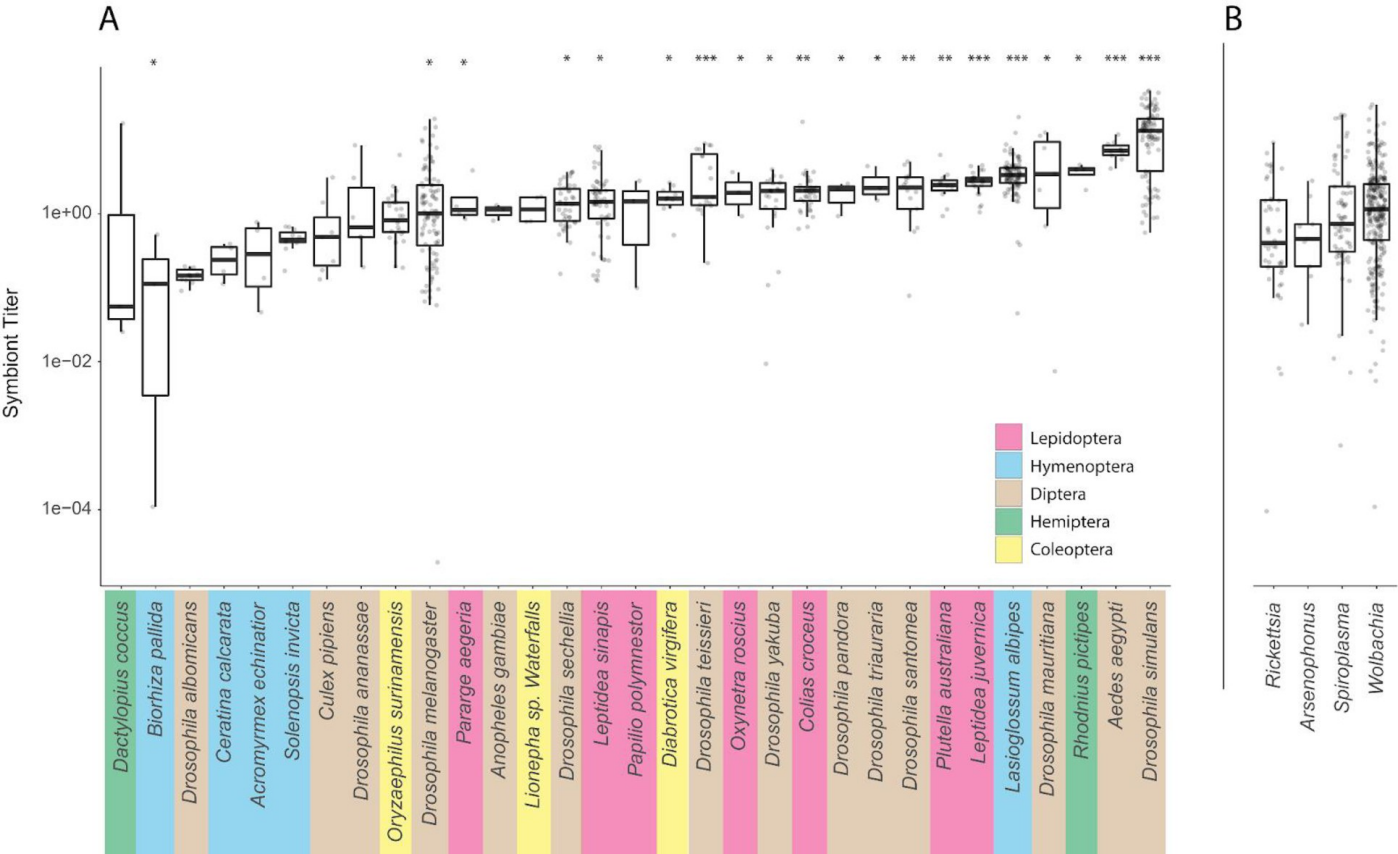
Titer variation across species infected with *Wolbachia* and between diverse reproductive manipulator clades. To increase readability of both plots, categories were randomly downsampled to show 100 samples. The y-axis is log10 scaled. **(A)** Titer for *Wolbachia* positive arthropod species with at least three samples were plotted from low to high titer and color coded by taxonomic order. **(B)** Titer for *Wolbachia*, *Rickettsia*, *Arsenophonus*, *Spiroplasma* infected samples. We plotted up to three samples for every species infected with *Wolbachia* to show the range of *Wolbachia* across tested arthropod species. Titer variation within host species is significant, and this variation is not due to pooled sequencing samples. Our results suggest a symbiont and host genetic contribution to shaping within-host infection densities. Asterisks indicate statistically significant relationships between titer and arthropod species where 0 ‘***’ 0.001 ‘**’ 0.01 ‘*’.

We found significant inter-sample variability in symbiont titer within host species (Fig 3A, S10 Table). For example, there is a more than 200-fold range in *Wolbachia* titer across *D*. *melanogaster* samples, consistent with previous estimates of titer in another *Drosophila* species [8]. Similarly, these results are in the range of previous estimates of *Wolbachia* titer in *Aedes Aegypti* [78]. To determine the degree to which host species influence variation in titer, we fit a linear model to the data. We found that *Wolbachia* titer in arthropods varied significantly between arthropod species (*p <* 2e-16, LM, Fig 3A), suggesting a strong genetic component shaping symbiont titer among arthropod species. Indeed, we found that this model can explain 36% of variation in titer. Moreover, we tested for the impact of *Wolbachia* phylogeny on titer differences by fitting a linear model with *Wolbachia* supergroup as a factor. We found that *Wolbachia* supergroups A and B explain about 1% of titer variation (*p =* 0.04). The remaining 63% of titer variation may be explained by within species differences, such as inter-individual variability and uncontrolled extrinsic factors, such as local diet or habitat. These results therefore support the idea that symbiont titer varies considerably both within and between host species.

Next, we tested for an effect of sample pooling on our results. As SRA metadata is not always complete, there is no way to be completely sure about the nature of the samples in the database. To confirm that pooling does not strongly affect estimated titer and therefore bias our results, we performed a literature search and annotated each sample as pooled or not pooled when this information could be confidently determined from the source publication (S11 Table). Then, we excluded samples that we could not categorize. We fit a LM model of the same form as above but with pooled sequencing status as an additional factor, we found that this model does not fit significantly better than a LM fitting titer and arthropod host species (*p* = 0.4, likelihood ratio test). This suggests the estimates from the whole SRA are robust, and that pooling samples does not significantly impact the variation we see in titer across arthropod hosts in this dataset. Our approach therefore appears robust to vagaries associated with aggregating diverse and distinct public datasets for computational analysis.

We hypothesized that *Wolbachia* titer in arthropod hosts could be significantly different compared to other reproductive manipulator bacterial titers because *Wolbachia* is found at substantially higher frequencies across arthropod species and many studies have reported that *Wolbachia* generally elicits smaller fitness effects on its hosts than do *Rickettsia*, *Arsenophonus*, and *Spiroplasma* [79–84]. Although an expanded model including reproductive manipulator as an explanatory variable provides a better fit to the data (*p* = 0.004, likelihood ratio test), this model explains only an additional 0.5% of the variance in symbiont titer relative to a model where only host species is included as an explanatory variable (see Methods). No reproductive manipulator clade significantly differed in contribution to titer variation (*p >* 0.05, likelihood ratio test). These results therefore indicate that titer does not systematically differ between clades of reproductive manipulators (plotted in Fig 3B), contrary to our expectations based on their infection frequencies and pronounced differences in their phenotypic effects. Finding that *Wolbachia’s* titer is relatively consistent with that of other reproductive manipulators suggests that co-evolved symbiont titer may not contribute to its widespread host range. Although, tissue-specific titers may vary among hosts and partially explain how fitness effects are mitigated. These results suggest that other co-evolutionary mechanisms, perhaps encoded by the host genome or regulating bacterial physiology, contribute to *Wolbachia*’s widespread taxonomic host distribution and generally decreased virulence relative to other widespread reproductive manipulator taxa [14, 85–87].

### The octomom region is associated with increased titer

The wMel genome contains a region of eight coding genes, referred to as Octomom, that when amplified in copy number is associated with increased *Wolbachia* density and virulence to the host [88]. We estimated the extent to which variation in titer is due to variation in Octomom copy number within *D*. *melanogaster* samples in the dataset (see Methods). Of the *D*. *melanogaster* samples, we found that 98% of them had two or fewer copies of Octomom (S11 Fig in S1 File). This is consistent with a previous study that found, on average, 1 to 1.5 copies of the octomom locus [77]. Using a linear model, we found Octomom copy number accounts for about 1% of the titer variation in *D*. *melanogaster* in the dataset. Our titer estimates are consistent with previous studies on Octomom titer [88]. This indicates that our method can find real genetic components of titer regulation in the wMel strain infecting *D*. *melanogaster*. Indeed, Octomom is certainly not the sole factor controlling symbiont density, and other determinants such as environmental effects and host genetics are associated with shaping symbiont titers [7, 76]. Nonetheless, these results present useful information on the broad scale dynamics of Octomom copy numbers across a variety of *D*. *melanogaster* samples from different labs, and provide evidence for low copy numbers of Octomom in *D*. *melanogaster*.

### Testing association between CI-loci copy number and infection prevalence

*Wolbachia* can rapidly spread by manipulating the reproductive system of its host, but manipulations are not required by all strains to reach intermediate frequencies [86, 87]. Cytoplasmic incompatibility (CI) is one of the most well-studied mechanisms of negative reproductive manipulation in *Wolbachia* and works by rendering host sperm and egg unable to form viable offspring [89, 90]. To assess the relationship between *Wolbachia* infection prevalence among arthropod hosts and the genetic capacity for CI, we leveraged the variable relative read depth at CI-inducing loci, *cifA* and *cifB*, among samples that were classified as *Wolbachia* positive, most closely aligned to wMel, and which had literature evidence for CI. Computing the depth of read coverage of *cifA* and *cifB* for each of these samples, we found no correlation between CI-locus copy number and host species infection frequency (*p>*0.05, S12 Table). However, given that the sample sizes for most species in our analysis are relatively modest, our power to detect an association is limited. Future studies with larger sample sizes will be able to build on our framework to address the fundamental connection between CI-causing loci and infection frequencies.

### Host genotypes associated with *Wolbachia* titer

Considering the significant influence *Wolbachia* strains can have on their hosts’ reproductive outcomes, it is likely that the host genome experiences strong selective pressures to respond to or control the bacteria. Indeed, previous work [7] identified a gene in the wasp host, *Nasonia vitripennis*, that effectively controls *Wolbachia* titer and has been under recent positive selection, likely since the wVitA strain horizontally transferred into the species. To address this question using our public data-sourced method, we used the *Drosophila* Genetic Reference Panel (DGRP) to perform an exploratory genome-wide association study (GWAS) of titer in *D*. *melanogaster* using the DGRP GWAS webtool [46]. Although the present analysis is exploratory, there are several noteworthy results that may suggest mechanisms of host control over *Wolbachia* titer.

We identified 10 candidate single nucleotide polymorphisms (SNPs) in the *D*. *melanogaster* genome associated with *Wolbachia* titer (S13 Table). We ran a permutation test on our dataset and found our FDR is 10%. One of the SNPs most strongly associated with titer variation is found in a gene associated with the endoplasmic reticulum (ER) membrane (*XBP1*) [91]. Consistent with this functional prediction, a recent study on a wMel-infected *D*. *melanogaster* cell line found that *Wolbachia* resides within ER-derived membrane near and within the ER itself [92]. Modifying this membrane might therefore enable the host to impact *Wolbachia* titer on the cellular level. Additionally, another SNP strongly associated with titer variation is found in a gene associated with actin binding and microtubule transport (CG43901). Recent work has shown that *Wolbachia* uses host actin for localization in host tissues [93, 94] and modifications to actin-binding proteins can clear *Wolbachia* infections in host individuals [95]. Finally, a SNP in CG17048 is also strongly associated with *Wolbachia* titer in the DGRP. This gene’s role in protein ubiquitination is consistent with previous results from a genome-wide RNAi screen that found alterations in host proteolysis contribute disproportionately to modifications of *Wolbachia* titer in cell culture and developing oocytes [96]. These are therefore appealing candidate genes for evaluating the potential of natural host variation to control symbiont infections.

These titer-associated genetic polymorphisms suggest that the host genome is capable of evolving to control *Wolbachia* infections. Future functional work will be necessary to validate our specific predictions. This present example is necessarily focused on a well-characterized genetic mapping panel in *D*. *melanogaster* due to data availability. However, our results illustrate more generally the potential impacts of high throughput data mining for identifying candidate host genetic factors that may be involved in controlling the infections within host tissues. This approach will become a more powerful tool as the data repositories continue to grow in host population depth and phylogenetic breadth.

## Conclusion

Here, we presented a reference-based approach to detect bacterial infections in publically-available short read data and highlighted the ways it can be used to generate insights into host-symbiont interactions. Indeed, our work is the first to estimate the global distribution of multiple reproductive manipulators across all sequenced arthropod and nematode hosts using full-genome high throughput methods. Moreover, we show that publically-available short read data can be used to interrogate other biological attributes of host-symbiont associations, such as titer, and elucidating candidate host genes undergoing natural selection. We found that symbiont titer, measured as ratio of symbiont symbiont genome complements to host genome complements, is highly variable among arthropod host species, and that titer can vary 200 fold between and within host species. Furthermore, our analysis detected novel infections among the vast phenotypic and genetic diversity of reproductive manipulators. These results could either be used directly to inform on useful biological control agents to control the spread of infectious disease or indirectly to inform on the natural infection state of host targets.

While our approach relies on datasets gathered for a wide array of purposes and therefore requires a level of approximation, we have shown that accurate and robust predictions can be obtained using this method. Moreover, as publicly available sequence data continues to accumulate at exceptional rates, this framework will become increasingly powerful relative to gathering purpose-built datasets to assay symbiont infection statuses and frequencies. More generally, our method and related approaches could be used to detect other microbial symbionts, such as medically relevant pathogens, or even viruses, for which a reference genome sequence is available. Hence, future work will build on this framework of leveraging increasingly vast datasets to conduct direct and precise hypothesis testing of fundamental questions in host and symbiont ecology and evolution.

## Methods

### Determining infection status using a BLAST-based approach

#### Reproductive manipulator reference genome panel

We built a BLAST database using RefSeq genome assemblies for *Arsenophonus*, *Spiroplasma*, *Rickettsia*, *Cardinium*, and *Wolbachia*. We included three *Arsenophonus*, six *Wolbachia*, 27 *Spiroplasma*, five *Cardinium*, and 61 *Rickettsia* genome assemblies (S14 Table). These genomes were selected to span the known diversity of these bacterial groups. We included comparatively more genomes from *Arsenophonus*, *Spiroplasma*, *Rickettsia*, and *Cardinium* because the genetic diversity in these groups is much less well characterized compared to *Wolbachia* and because fewer positive controls are available. We therefore sought to identify the largest number possible. On average, each genome assembly was about 1.3 Mb. We used these 102 genome assemblies to build a BLAST database using the blastdb command from the NCBI blast package (version 2.7.1). We also included additional *Wolbachia* reference genomes to estimate symbiont titer, which are listed in S14 Table, bringing the total number of references to 137.

#### Arthropod and nematode SRA dataset

We downloaded all Arthropoda sequencing read data from the NCBI Sequence Read Archive (SRA) database [44]. We filtered samples under the Arthropoda and Nematoda taxonomy for those sequenced on Illumina sequencing platforms. We filtered nominally for whole genome shotgun libraries, but for completeness we further removed samples that were marked as “reduced representation”, “chipseq”, and other terms that preclude a fully random, shotgun library approach. In total, we tested 27,256 arthropod and 5,229 nematode samples for reproductive manipulator infections. We consolidated all subspecies into a single species which resulted in a total of 1,299 arthropod and 128 nematode species. SRA metadata for samples we classified can be found in S15 and S16 Tables. Note, multiple sequencing runs may belong to one biosample.

#### Downloading reads from arthropod and nematode samples

First, we used fastq-dump 2.9.0 from the SRA Toolkit to download all reads from each sequencing run associated with each sample. For example, if a sample was sequenced in three runs, we would use six million reads to classify the infection status of that sample. An example of our command line is shown below.

$ fastq-dump—fasta—split-files -I—stdout -X 1000000 ERR1882042.sra > ERR1882042.fasta

#### Computing summary statistics

Next, we aligned these reads to the set of reproductive manipulator genomes using blastn (version 2.7.1). We computed three summary statistics to help describe the similarity between sample reads and reproductive manipulator reference genomes. These statistics were: 1) the breadth of coverage, 2) the variance coefficient, and 3) the estimated depth of coverage, and are described below. To calculate these statistics, we first divided each reference genome into non-overlapping 5kb bins, and we placed reads into bins based on their reference match start position. If a read aligned to multiple places in a reference genome, only the match with the lowest e-score was considered.

We computed all summary statistics for each reference independently. First, we computed the breadth of coverage as the proportion of 5kb bins with at least one read. Second, the variance coefficient was computed as the mean variance of the number of binned reads across 5kb bins, normalized by the total number of reads in all bins. Lastly, we estimated the depth of coverage by using the total base pair length of significant BLAST hits generated from our pipeline, the total number of possible reads in a sequencing run, and the number of reads sampled using our pipeline. Specifically, we used the fraction of BLAST base pair matches within the number of sampled reads to solve for the expected number of symbiont base pair matches given the total number of reads available in a biosample. We divided the expected length by the symbiont reference length to compute the estimated symbiont depth of coverage within a biosample.

#### Criteria for positive infection

In order to classify a host sample as infected or uninfected with a reproductive manipulator, we analyzed local alignments between host DNA sequence data and reproductive manipulator reference genomes. We binned each reference genome into 5kb segments and computed the proportion of bins with a significant hit (breadth of coverage). We also computed the variance of BLAST hits across each bin and estimated the coverage of the reproductive manipulator genome. We determined a sample to be a candidate for a positive infection if it had a 90% breadth of coverage and >1x estimated coverage on a reproductive manipulator reference genome. If a sample had between 0.1 and 0.9 breadth of coverage, the full dataset was downloaded and the workflow repeated to update predicted infection statuses. This two-step procedure allowed us to substantially decrease runtimes, because 97% of samples can be categorized using just 2 million read pairs, and to avoid false negatives associated with low sequence coverage positive infections.

### Validation of bacterial detection pipeline

#### *Wolbachia* infection status of the DGRP

We determined the reproductive manipulator infection status of 158 individuals from the *Drosophila Genetics Research Panel* (DGRP) [46] using our computational pipeline. Two samples within the DGRP were removed from our dataset because they contained conflicting PCR and WGS classifications. These two samples were classified positive for *Wolbachia* from PCR analysis, but were classified negative using a previous WGS analysis [41]. This conflicting infection status could have been caused by PCR sample contamination and subsequent false amplification of a *Wolbachia-*specific fragment. Similarly, low-level contamination during library preparations could result in false positives using our bioinformatic approach. However, this would have to be a significant amount of contamination, as fewer PCR cycles are run with general Illumina primers in most Illumina library preparation protocols. We removed these two samples from our dataset, thereby only using samples where WGS and PCR classifications were concordant in previous studies.

### Beta-binomial estimation of reproductive manipulator frequency

#### Beta-binomial rationale

Our study aims to directly sample and determine the infection statuses of more animals of any study to date. However, there is an inherent sampling bias in any study that tries to estimate the frequency of reproductive manipulators within species where infection might occur at intermediate or low frequencies. For example, the probability of sampling one individual from a population with high infection frequency (i.e. many individuals are infected) is higher than sampling an infected individual from a population with low infection frequency. The probability, then, of classifying a population as infected is dependent on the number of individuals tested and the frequencies of infection within each species. To evaluate and correct for this ascertainment bias, we use a beta-binomial distribution to estimate the total proportion of reproductive manipulator infected species.

Beta-binomial and double inflated beta-binomial distributions have been used to fit *Wolbachia* infections previously [34, 35]. We fit both models to real and simulated data to determine which model best describes our data. We simulated a dataset where alpha = 0.07, beta = 0.5, phi = 0, gamma = 0, where the success of drawing a given count of infected individuals is a function of the beta-binomial distribution with parameters independently drawn for each species, and the number of individuals drawn is defined by n, the number of individuals samples in our real dataset. Using the likelihood models described in [35], we found the double inflated beta binomial model did not fit significantly better to real or simulated data than the beta-binomial. Thus, we chose the simpler two-parameter beta-binomial model to model our data. The beta-binomial model considers N random variables, X_j_, which are all binomially distributed (*i*.*e*., infected vs. not-infected), but each with different parameters q_j_ and n_j_, so that X_j_~Bin(q_j_, n_j_) (S12 Fig in S1 File). Using the approach developed in [34], we determined (1) moment estimators *u* and *s*, (2) beta distribution parameters α and ∝, and (3) the global infection rate *x*. After we fit a beta distribution to the data, we took the integral from *c* to 1, where *c* is the minimum infection rate of a species to be considered positively infected. For example, a value of 0.001 means a species would be classified as being positively infected if one individual in 1000 is classified as positive. Indeed, low frequency infections in species would be hard to detect with much precision yet can impact frequency estimates using the beta-binomial distribution. We estimated global infection frequencies using different *c* thresholds (S17 Table). For consistency with previous work, we set *c* to equal 0.001 (as in [34]). Additionally, see sections below for downsampling results, which aimed to reduce sampling intensity bias.

#### Mitigating sampling bias by downsampling

We tested whether pruning our dataset was necessary to estimate the global infection frequency of reproductive manipulators in arthropods and nematodes. Since the beta-binomial model is positively influenced by the number of species, and number of individuals sampled per species in the dataset, Hilgenboecker tested a variety of downsampling methods in order to mitigate the influence of a non-uniform sample set would have on their global estimates [34]. In addition, Hilgenboecker gathered their infection status data from studies measuring the prevalence of *Wolbachia*. The authors state that curating a sample set from these studies almost certainly introduced bias towards finding *Wolbachia* in a sample, as the studies focused on species known, or at least suspected, to have *Wolbachia*. Our method contrasts with this sampling approach, and implements a more random strategy to sampling arthropod and nematode species. Although there is still a sampling bias to what species are sequenced (i.e. medically relevant, model systems), our approach does not specifically bias toward species that are thought to harbor reproductive manipulators (S18 Table).

To test the effect of non-uniform sample size distribution among host species, we fit beta-binomial models to downsampled datasets. To downsample, we chose a maximum threshold (nj_max) in which all species would be downsampled to have a maximum of nj_max individuals. To downsample individuals within a species to nj_max, we randomly chose individuals without replacement from within the species. Confidence intervals were computed via 1000 bootstraps replicates.

With the full dataset, the *Wolbachia* global infection frequency 95% confidence interval is estimated to be between 0.2 and 0.7 (S13 Fig in S1 File). Estimates of *Wolbachia* global infection frequency decreased as datasets were downsampled. Moreover, we see the 95% confidence interval become tighter around the mean as we downsample the original dataset (S12 Fig in S1 File). These results taken together suggest that the beta-binomial model is influenced by the few large sample sizes in the dataset and varying global infection frequencies can be produced depending on the data set used. Nonetheless, we see a stable global infection frequency of *Wolbachia* around 0.4 when species are downsampled to 100 individuals. Therefore, we computed global infection frequencies using a dataset downsampled to 100 individuals per species.

#### Estimating symbiont titer

We used DNA reads to estimate the ratio of symbiont genome complements to host genome complements, hereafter referred to as titer. We estimated the number of symbiont genomes by BLAST-ing sequencing reads to a *Wolbachia* reference database and estimating depth of read coverage (see subsection *“Computing summary statistics”* above). We used a set of single copy orthologous proteins from arthropods using OrthoDBv9 to estimate the density of host cells in a sample. This set contained 1066 proteins, from 133 taxonomic groups spanning Arthropoda, which amounted to a total 312,654 amino acids of reference sequence [97]. We used a tblastn based approach to locally align host nucleotide reads to the arthropod orthologous protein sequences. Because not all arthropod single copy ortholog proteins might be present in the host sample, or because there might be regions of the protein that are divergent from the host sample, we used the average of maximum depth across orthologous proteins that have reads mapping to them as an estimate of host titer. We corrected for the ploidy difference between host (diploid) and symbionts (haploid) by halving the computed host coverage to result in a titer estimation (symbiont haploid: host pseudo haploid).

We confirmed that our tblastn approach is consistent with a whole genome reference-based alignment approach when computing host genome coverage. For reference based alignments, we chose samples from ten different arthropod clades representing arthropod diversity of which there was a known host reference genome. We used BWA-MEM to align sample reads to the reference genome and computed average depth of coverage across all sites using samtools depth. We then compared the depth of coverage computed from the reference-based approach to our ortholog tblastn. These results corresponded well to the results from our ortholog approach, so we continued with the previously described workflow.

Furthermore, titer estimates might be impacted by the distance to the reference selected. We would expect symbiont coverage estimates to vary significantly between references if titer estimates were impacted by the distance to the reference selected. We investigated potential reference bias within our titer estimation by varying the symbiont reference selected. For each positively infected *Spiroplasma*, *Rickettsia*, *and Arsenophonus* samples in Fig 3, we compared the first and second symbiont reference genomes with the highest depth of coverage. We did this for all 132 *Spiroplasma*, *Rickettsia*, and *Arsenophonus* samples in Fig 3 and found no major differences in depth of coverage between the two references (S9 Fig in S1 File). For *Wolbachia*, we similarly compared the first and second *Wolbachia* reference genomes with the highest depth of coverage, and also aligned the sample to the *Wolbachia* reference genome to test the accuracy of our method. We computed a depth ratio between the estimated depth of coverage from our BLASTn approach with a reference based approach and found no significant difference among this ratio between the closest and second closest *Wolbachia* reference (S9 Table). This suggests that our method is robust in estimating stable symbiont genome coverages especially from *Wolbachia* Supergroups A and B, of which comprise the majority of tested arthropod infections.

#### *Drosophila* oocyte sampling, imaging, and analysis

We obtained *Drosophila melanogaster* and *Drosophila simulans* fly stocks infected with the wMel and wRi strains of *Wolbachia* from William Sullivan’s Lab at UCSC. Stage 9/10a oocytes were dissected from these flies, stained and mounted on glass slides, and imaged with a SP5 Leica confocal microscope. We analyzed the fluorescence due to *Wolbachia* as described in [26].

#### Markers and balancers

The *D*. *melanogaster* stocks used were carrying the markers and balancers w[1]; Sp/Cyo, Sb/Tm6, Hu or the germline double driver: P{GAL4-Nos.NGT}40; P{GAL4::VP16-Nos.UTR}MVD1. These stocks were infected with the wMel strain of *Wolbachia*. The *D*. *simulans* stock used was w[–] and was infected with the wRi strain endogenous to *D*. *simulans* populations in North America.

#### Collection

Flies were collected shortly after eclosion and transferred to new white food for three to five days. We dissected the ovaries of approximately 10 flies of each species in 1xPBS, and “fluffed” the ovarioles with pins to separate them. The ovaries were fixed in formaldehyde and heptane, RNAse A treated, and stained with propidium iodide, as described in [26].

#### Imaging and analysis

We mounted the stained oocytes on glass slides and imaged them with a SP5 Leica confocal microscope using a 63x objective. We imaged oocytes through their middle planes, taking optical sections every 0.38 um, the Nyquist value. For analysis, we picked comparable planes approximately halfway through the oocyte for all imaged oocytes, and created 3D brightest point projections from three slices, representing the diameter of one *Wolbachia* cell (~1 μm), in ImageJ. We analyzed these images for fluorescence due to *Wolbachia* by manually removing fluorescence due to host cell nuclei, thresholding the image to eliminate background noise, and measuring the fluorescence contained within the entire oocyte cyst as described in [26]. We calculated the corrected total cell fluorescence (CTCF) for each cyst with the following formula: CTCF = Integrated Density–Area of selected cell X Mean fluorescence of background readings. Results were plotted with the vioplot package in R.

### Identifying previously unreported infections

We searched the literature to identify previously unreported *Wolbachia*, *Spiroplasma*, *Rickettsia*, and *Arsenophonus* infections in the SRA scan dataset. We used Google Scholar, and searched [species] + [reproductive manipulator name]. If no published results were found, we also searched the results of [37] who did an SRA scan, albeit smaller than ours. If no infection was found using both these methods, a species was determined to be a novel infection. Our method to determine novel infections is not exhaustive, especially if we consider species nomenclature can change over time. Nonetheless, these candidate novel infections illustrate the potential impacts of our method.

### Co-infection permutation test

To test whether our observations of co-infected species, where one species harbors observations of two or more reproductive manipulator strains, exceeded what would be expected by chance, we conducted a permutation test. We also performed a similar test for individual-level coinfections, where we asked if the number of individuals that were coinfected in our data exceeded what would be expected by chance. To do this for each species and for each reproductive manipulator we drew a probability, *p*, from the beta-binomial distribution of each reproductive manipulator infection frequency. Then, for each species, we drew samples from a binomial distribution with parameters *p* and *n* where *n* is downsampled to a maximum of 100 for each species for consistency with our analyses. We counted the number of individuals with co-infections as well as the number of species with co-infection and reported a *p-*value based on how many of the 1000 bootstrap replicates were greater than or equal to our observed counts (S5 and S6 Figs in S1 File). This approach is preferable to an individual-based permutation that ignores the frequency of each symbiont within the population because it controls for the autocorrelation among individuals within a population by requiring that the infection frequency be fixed for each population or species. It is therefore unaffected by differences in sample sizes among species.

### Estimating the biological and methodological contributions to titer variation

#### Host/symbiont genetics

We fit several models to determine how much host/symbiont genetics contributes to variation in titer. We fit Linear Models models to log transformed arthropod titer data using the glm() functions in R version 3.5.0. Specifically, we fit a LM of the form: glm(log(titer) ~ arthropod_species). We also compared that model to one including reproductive manipulator clade (e.g. “*Wolbachia*”) using a LM of the form: glm(log(titer) ~ arthropod_species + reproductive_manipulator_clade. We used a likelihood ratio test to compare these two model fits using anova(model1, model2, test = “Chisq”).

#### Pooled sequencing and tissue sampling

Variation in tissues and pre-sequencing treatment of samples could affect symbiont titer levels. Additionally, pooling several individuals for DNA extractions might also impact mean titer estimates if infections are polymorphic within species. Considering this bias, we reviewed and aggregated the methods used to sequence samples in our comparative study of titer among symbiont species (Fig 3, S11 Table). We used the project accession numbers found in the SRA metadata to trace back to primary literature describing the origin of each sequencing dataset. We report and retain samples that we could confidently categorize as pooled on not-pooled. To test for an effect of sequencing strategy, we fit a LM of the form: glm(log(titer) ~ arthropod_species + pool_status) to samples where whole adult individuals were sequenced and did not have specific environmental, chemical or dietary treatment (See “Comparative Study of Titer Across Symbiont Taxa” in Results)

All samples that were positively infected with *Arsenophonus* appear to be pools of whole adult individuals based on our literature review. The samples we tested for Arsenophonus were pooled from about 10 individuals, which means *Arsenophonus* titer might be greater than what we present, if *Arsenophonus* infection frequencies were less than one within host populations. Given that the titer data shows titer varies with orders of magnitude between symbiont strains and given that *Arsenophonus* samples were in small pools, we would expect unpooled *Arsenophonus* samples would fall within the variation of symbiont titer, however additional unpooled samples are necessary to precisely describe the titer of *Arsenophonus* within arthropod hosts.

### Octomom copy number estimation

We tested the hypothesis that Octomom loci amplification in the wMel genome increases symbiont titer within a host individual [88]. For infection-positive *D*. *melanogaster* samples in our dataset, we computed the depth of coverage using bwa-mem and samtools depth in the Octomom region of wMel (488,974–507,200 bp, ASM802v1). We then randomly divided non-octomom sites into two sets, the depth of coverage between the two non-octomom sets of loci were highly correlated (rho = 0.99, Spearman). We generated two sets of non-octomom sites in order to mitigate interdependence between computed values and any linkage between sites adjacent to the octomom region. One set was used to compute symbiont titer and the other was used to compute the octomom copy number. Host depth of coverage was computed like previously described. We fit a LM to octomom copy number data in the form GLM(symbiont_titer ~ octomom_copy_number).

### Testing association between CI-loci copy number and infection prevalence

We tested the association between known CI-loci in wMel (cifA and cifB) and host species infection frequency [89]. We selected samples from the dataset that have evidence for CI-phenotype, and generated the highest breadth of coverage when aligned to the wMel reference genome during classification. We aligned sample reads to wMel reference genome and computed the read coverage genome wide, within the cifA region (617,223–618,647bp), and within the cifB region (618,702–622,223bp) (S12 Table). We tested for association between CI-loci copy number and host species infection prevalence using a Spearman’s correlation.

### DGRP genotypes associated with *Wolbachia* titer

We used the DGRP GWAS tool to obtain SNPs associated with *Wolbachia* titer [46]. The GWAS was conducted on DGRP flies that were raised in a single lab with tightly controlled conditions. Manhattan plots and QQplots generated from the GWAS are available in S14 Fig in S1 File. We obtained an empirical FDR by permuting the strains and titers of our data matrix, and computing the number of SNPs that would be considered significant given p-value cut-offs (S19 Table). We chose a p-value cut-off of 3.11E-07 that minimized the FDR. We computed the empirical FDR computing the number of SNPs identified at or above the p-value cut off in the permuted and real datasets, and then dividing the permuted by the real value.

## Supporting information

S1 Data Positive arthropod classification data. (XLS)

S2 Data Positive nematode classification data. (XLS)

S3 Data All arthropod classification data. (TXT)

S1 File (DOCX)

S1 Table DGRP infection classification accuracy. (XLSX)

S2 Table DGRP infection classification accuracy. (XLSX)

S3 Table Classification of Pascar samples. (XLSX)

S4 Table Classification of divergent *Wolbachia* strains. (XLSX)

S5 Table Arthropod SRA scan species infection counts. (XLSX)

S6 Table Nematode SRA scan species infection counts. (XLSX)

S7 Table Novel infections in arthropod species. (XLSX)

S8 Table Arthropod species with co-infections. (XLSX)

S9 Table *Wolbachia* titer validation. (XLSX)

S10 Table Symbiont titer estimates. (XLSX)

S11 Table Pooled status of arthropod samples. (XLSX)

S12 Table CI-loci association study. (XLSX)

S13 Table DGRP titer GWAS results. (XLSX)

S14 Table Symbiont references metadata. (XLSX)

S15 Table Arthropod SRA metadata. (XLSX)

S16 Table Nematode SRA metadata. (XLSX)

S17 Table Varying c threshold experiment. (XLSX)

S18 Table Infection bias comparison to Hilgenboecker. (XLSX)

S19 Table Permutation results of GWAS SNPs in DGRP associated with titer. Results are sorted by p-value.
(XLSX)
